# Re-storying health promotion models toward population sovereignty

**DOI:** 10.3389/fpubh.2026.1771109

**Published:** 2026-03-26

**Authors:** Kimber Olson, Matt Erb, Emily Si’al, Alta Piechowski, JoAnne Riegert, Noshene Ranjbar

**Affiliations:** 1 Juniper Pine Consulting, Anchorage, AK, United States; 2 EARTH: Empowering All Relatives to Heal, Tucson, AZ, United States; 3 Independent Scholar, Tucson, AZ, United States; 4 College of Nursing, University of Arizona, Tucson, AZ, United States; 5 San Francisco Tlingit and Haida Community Council, Sacramento, CA, United States; 6 The Hozho Voices of Healing Center, Crownpoint, NM, United States; 7 Ombimindwaa Gidinawemaaganinaadog White Earth Elder, Waubun, MN, United States; 8 Department of Psychiatry, University of Arizona College of Medicine-Tucson, Tucson, AZ, United States

**Keywords:** salutogenesis, indigenous, health promotion, decolonization, mind-body medicine, health sovereignty, traditional indigenous knowledge

## Abstract

Traditional Indigenous Knowledge, Culture, and Practices (TIKCP) underpin the emergence of contemporary salutogenic and integrative health models, yet Indigenous voices, teachings, and perspectives often go unrecognized in the development, dissemination, and spread of these contemporary models. Re-storying—cultural reclamation within a complex sociocultural landscape—is growing as a concept and approach that supports healing and sovereignty in populations adversely impacted by dynamics of oppression. Situating these efforts within TIKCP demands repeated evaluation and inclusion of their unique historical, sociocultural, and sacred origins. Without this undertaking, historical and ongoing injustices, power imbalances, and cultural appropriation can be perpetuated, further threatening the self-determination. Healing within and between sub-populations, through epistemic and cultural humility, is supported when respect for TIKCP is openly acknowledged, explored, and supported during dissemination of modern versions of ancient systems. This community case study draws upon a specific contemporary salutogenic healing model to explore how intentional processes of reconciliation and Indigenization can be approached to support healing, well-being, and health sovereignty. Fostering respect for and honoring Indigenous experiences and voices supports culturally attuned environments that in turn amplifies potential for individual and community-wide well-being. The framework described for re-storying a westernized and evidence-based model of mind–body medicine carries potential to support healing from historical trauma, ambiguous loss, and ongoing harm linked to colonialism, present-day prejudice, injustice, and discrimination.

## Introduction

### Background

While variable across global subpopulations, there is a set of principles underlying Traditional Indigenous Knowledge, Culture, and Practices (TIKCP) that inform Indigenous worldviews, including those of the health-illness spectrum. TIKCP encompasses the belief systems, scientific frameworks, language, customs, traditions, ceremonies, and collective practices that arise from generations of relational, place-based knowledge exquisitely interwoven with the natural world. In historical context, the dramatic changes of the last few centuries, combined with the colonial ideology of superiority and domination, set up sociocultural suppression and diminishment of the value of TIKCP. Many “modern” technologies, theories, and emerging health frameworks, while housed in contemporary scientific frameworks, have roots in the world’s wisdom traditions that preceded the western model of inquiry. Here, salutogenesis as a contemporary construct of health promotion and a specific salutogenic-minded healing model will be explored in relation to TIKCP.

Indigenous peoples of the Americas are the focus of the lived experience of the authors. We aim to present this experience alongside an adaptable framework that can be explored within other Indigenous communities such as those located in India, Finland, Australia, Russia, Africa, etc. Regardless of geographic locale, Indigenous populations, along with their knowledge systems, are centered throughout this document by capitalizing terms such as Tribe, Nation, and Indigenous, while intentionally using lowercase for terms like western and westernized to “disrupt the privileging of these terms” (1, p. 359). “These words and concepts have been emphasized enough. They are often unintentionally centered because we remain largely unaware of their neurobiological and linguistic hold on us, even when we recognize the social implications” (2, p. 7).

In this undertaking, there is risk of reinforcing the duality of good vs. bad or right vs. wrong, which would paradoxically violate TIKCP principles. As such, approaching this discourse with the aim of balance and psychological flexibility that recognizes co-existing utility and limitations of various worldviews is encouraged. In Indigenous-led scholarship, much has been written about this, including around the concept of *etuaptmumk*—a word in the Mi’kmaw language that roughly translates as two-eyed seeing. Etuaptmumk infers learning to simultaneously see and act with the strengths of Indigenous knowledge and ways of knowing alongside the strengths of western knowledge and ways of knowing (3). This concept does not reduce viewpoints to binary thinking. Instead, we are encouraged to realize the multiple identities each of us holds and to think in terms of multiplicity. Multiple adaptive pathways and contextually attuned interventions are implicit within a TIKCP framework. Recognizing universality (e.g., humans everywhere experience emotion) within a non-uniformity of expression (e.g., “openness” in a western context may mean expressing feelings, while “openness” in an Indigenous concept could mean maintaining harmony) helps demonstrate psychological flexibility within the limited discourse of conceptual frameworks. Shared human capacities are expressed differently across the multiplicity of identities, even within a single individual. We are promoting a non-binary view toward a framework for co-creating customized pathways for health promotion that more optimally serve the unique, place-based needs of sub-populations within the umbrella of Indigenous populations.

### Origins of “salutogenesis” as a contemporary model of health

Medical sociologist Aaron Antonovsky posited salutogenesis in the 1970s. Antonovsky partially formulated his ideas from observations of how some Jewish Holocaust survivors stayed healthy despite having experienced life-threatening traumatic exposure under conditions of genocide (4). Salutogenesis has been integrated into the field of health promotion to examine determinants of health and well-being, and stands in contrast to pathogenesis, which focuses on illness and disease. Salutogenesis seeks to identify resources that help individuals and populations cope with stress and suffering; ultimately to live well despite life’s vicissitudes. This endeavor must be led by the voices of those being supported, and any outsiders must embody epistemic and cultural humility. *Epistemic humility* reflects consistently recognizing and acknowledging the limits of one’s knowledge alongside the demonstration of clear and authentic openness toward understanding and responding to new information and perspectives (5). *Cultural humility* reflects commitment to a lifelong practice of self-reflection that emphasizes understanding one’s own identity while deeply respecting and learning from the identities and lived experiences of others (6). Cultural and epistemic humility synergistically focus on reducing power imbalances and fostering mutual respect and understanding. These asset-based mindsets recognize and nurture the strengths of those being supported, including those strengths rooted in cultural wisdom and intergenerational knowledge (7).

While honoring the value and creativity of the salutogenic model, it can be argued that the embedded ideas were not “new.” Indigenous peoples have long lived with the worldview that the health of the environments (land, water, animals, plants, etc.) that they live in is intimately intertwined with their own health; they carry a sociocultural structure and set of related practices (TIKCP) that innately support well-being. The preceding underscores the innate place-based nature of Indigenous communities whose ways of life evolved in intimate relationship with local conditions. Prior work has explored the compatibility of salutogenesis alongside emancipatory and place-based pedagogy to a specific Indigenous (Anishinaabe) population (8). Here we explore the concept that TIKCP as a holistic framework for life precedes and exemplifies the principles of salutogenesis, even though Indigenous populations did not have it packaged in the same cognitive framework.

### Dominant culture influences health promotion design

Antonovsky explored stress and coping; his work built upon earlier western academics, most of whom were entrenched in the biomedical model (9). While western medicine has been generally effective at locating sources of illness within the body, it has largely avoided addressing sociocultural causes. Research increasingly shows that the conditions that people are born into and live with—despite capacities and choices—largely shape the life and health conditions that they experience (10). The growth of social determinants of health and epigenetics as critical public health topics is a direct reflection of the need to address “upstream” influences on health. Critical studies point out that we are wrong to see disease as residing solely in the body of individuals, and thus subject to individual biology, lifestyle, choice, and self-care (11). The belief that ill health arises solely from individual choice, genetics, or fate, and not from societal conditions, has become intertwined with the political and economic ideology known as neoliberalism. On the surface, neoliberalism advocates for free-market capitalism. However, hidden within this stance lies the biopolitics of agency, where deflection of responsibility for the collectivist dynamics that inform health, economic security, and more, resides. By focusing solely on the individual in isolation, we only address a small part of the problem, leaving the adverse health impacts (on Indigenous and other populations) that arise from the nature and design of systems, policies, and practices unexamined.

Scholars in these fields point out that chronic disease and compromised well-being are located within the economic, social, and political arrangements of capitalism and colonialism. It is argued that the structure of society significantly contributes to the conditions that give rise to ill health; the same structures then create and control a large variety of professions and systems to keep the effects that arise from it in check, often while deflecting attention away from its own contributions and profiting from it by maintaining socioeconomic power imbalances (10–13). Specific to Indigenous population, “Overwhelmingly, neoliberalism has manifest impacts, through various pathways, on poor health outcomes and experiences for Indigenous communities…” (14, p. 1).

How does this setup apply to pathogenesis and salutogenesis? Pathogenesis examines ill health and works backwards to determine how individuals can avoid, manage, or eliminate the condition. Research into social determinants of health is making it easier to identify causal relevance, though not necessarily effective solutions. For salutogenesis, the relevance may seem less obvious; it is important to consider that the concept partially arose out of a growing need to address the very problems that western societal structures produce. As such, even salutogenic and integrative approaches benefit from examining the degree to which they reinforce neoliberal-minded individualism (15, 16) that is not aligned with the collectivist-orientation of TIKCP. To re-story these models, recognition of the preceding historical, sociocultural, and biopolitical context is needed. This task is followed by efforts to support healing of intergenerational trauma alongside leveraging individual and community health assets which are defined as the sum of resources that enhance the ability of peoples to experience health and well-being while contributing to a reduction in health inequity (17). Both pathogenic and salutogenic approaches warrant ongoing advocacy for systemic change to truly support health and well-being.

## Context

### A science-backed model of well-being

Here, we explore a contemporary model of health promotion that utilizes mind–body medicine practices and that draws from a variety of wisdom traditions from around the world. This salutogenesis-oriented model has been carried forth into worldwide service across multiple Indigenous contexts by The Center for Mind–Body Medicine (CMBM). There is published evidence on this model’s usefulness for supporting challenges such as trauma, depression, burnout, and in support of overall well-being (18–21).

The mind–body skills group (MBSG) approach centers around strength-focused and healing-centered engagement; it includes ancient and multi-cultural practices such as group sharing, meditative awareness, creative arts, imagery, supporting personalized spirituality, and movement. The approach weaves in scientific understandings of toxic stress and trauma to create a cross-culturally deployable approach that speaks to the universality of core human tendencies and needs.

Consistent with honoring the contemporary creativity and utility of salutogenesis, the MBSG model underscores the importance of supporting foundations of health and resilience. Concurrently, the re-storying of this model toward the goal of redressing historical trauma and injustice done at the hands of colonialism is presented. Re-storying as a concept involves concurrent support for individual and community healing toward the larger goal of restoring the layered (social, cultural, economic, political, etc.) sovereignty of peoples and communities within the realities of modern life. While storytelling (known as narrative medicine within the biomedical model) is important within TIKCP as part of oral tradition and knowledge transmission, and is supported within the MBSG, re-storying is distinguished as part of larger proactive social justice movements.

Starting in 2009, two CMBM faculty members were invited to visit several Alaska Native communities, including Inupiat and Unangax, to offer mind–body medicine workshops. Subsequently, a CMBM trainee with ties to the Oglala Lakota community of Pine Ridge, South Dakota recognized the potential of the approach to complement this community’s existing health resources. A discussion was held with a local Elder and traditional healer around the TIKCP parallels. Several years of volunteer programming ensued, followed by philanthropic funding to support larger-scale professional training across the Pine Ridge Indian Reservation, as well as a 3-year Administration for Native Americans grant (US Department of Health and Human Services) to integrate the approach into schools. Over time, more than 800 members of and allies from and/or serving over 30 Native Nations have received training in the approach. The effectiveness of this approach was gaged based on the lived experience of the authors, qualitative feedback, observations of ongoing utilization by trainees, and continued interest in receiving training. These efforts have also revealed vital insights into the challenges of the approach. These challenges can arise when program delivery bypasses organically building deep relationships and rapport with the community. In addition, acknowledging a community’s unique culture and sovereignty is vital to establishing the tone, intention, and mechanisms of delivery. Many of the challenges encountered in this work can be tied back to the history and ongoing adverse effects on Indigenous populations from colonialism, capitalism, extractivism, attempted genocide (including of the cultural domain), and contemporary neoliberal ideology. In noting that the concept of success is not absolute in context to TIKCP, as well as the understanding that hard data is valued differently, we have found that the best measure of relative success is found in the level and depth of engagement of community members. Healing and reclamation of sovereignty is a complex ongoing process that requires cycles of feedback– each community is invited to gage value and develop their own measures of success.

Most recently, a Chiricahua Apache citizen experienced with the MBSG explored Indigenous re-storying of the approach as part of a doctoral degree in grief counseling (2). Drawing upon this work, and the lived experience of the authors who have worked with CMBM’s (and other) collaborative programs with Indigenous populations, this article further delineates rationale and strategies toward the ongoing re-storying of health promotion models in partnership with Indigenous knowledge keepers. With this partnership, reciprocal integration of modern neuroscience with traditional Indigenous healing practices is possible. We present a framework that aims to be flexible for adaptation across globally distributed Indigenous sub-populations.

### Lived experience of indigenous populations

Before presenting further details of re-storying the MBSG model, it is vital to recognize the ongoing impacts of colonial history on Indigenous persons. Since first contact, colonial practices have sought to deprive Indigenous people of access to their ways of knowing, being, and doing. Indigenous people experience pervasive disparities stemming from centuries of attempted genocide (22). These disparities continue to manifest as individual, collective, intergenerational, and historical trauma, along with ambiguous loss. Practices tied to colonialism have led to ongoing cultural genocide and trauma for Indigenous communities (23). These factors contribute cyclically and intergenerationally to high rates of suicide, alcohol and drug abuse, child abuse, physical violence, depression, and sexual violence (24–26). The resulting challenges interact and are synergistically interrelated and deeply connected to land loss, the destruction of sustainable ecosystems (27, 28), insufficient nutritional and traditional food sources (29, 30), and access to adequate health (31) and behavioral health (32) services. Complicating this, and specific to mental, emotional, and spiritual well-being, mainstream western psychology was founded on a white, middle-class, eurocentric perspective based on research almost exclusively conducted with WEIRD (western, educated, industrial, rich, democratic) populations and communities (33, 34). When Indigenous perspectives, beliefs, value systems, and treatment methodologies were (rarely) utilized by western health systems, they were typically misbranded, misattributed, or outright appropriated (stolen).

To address this ongoing harm, persons working with Indigenous populations, and the larger whole of western society, must free their minds, bodies, and organizational systems of misconceptions, biases, and practices that perpetuate harm. Per the principles of cultural humility, this is not a singular effort, rather a lifelong process requiring open feedback loops and personal introspection. Individuals and organizations must be introduced to effective frameworks and practices that guide acknowledging, understanding, and acting on Indigenous perspectives in support of healing and well-being. These efforts must include openness toward community-led modification alongside integration of traditional practices without attempts to admonish or control the process (2).

The best therapeutic practices of healing are demonstrated by first knowing oneself, followed by understanding the community and the individuals being served (35–38). Knowing the history of Indigenous colonization is inadequate to become a competent consultant, advocate, or ally. Even a broad understanding of colonization in relation to contemporary sociocultural dynamics fails to address the unique strengths and traumatic histories of specific communities, families, or individuals. Cognitive understandings alone also typically fail to adequately recognize, support, and integrate the depth and utility of existing restorative practices, spiritual ceremonies, and healing rituals that are known by and accessible to community members. This is essential knowledge for anyone looking to partner with Indigenous peoples in their healing process (2).

Psychologist, healer, and author Ruby Gibson, PhD, shared in a conversation (June 1, 2023) that “Trauma happens when we lose our voice, choice, and our power. Healing happens when we reclaim those”. If we aim to positively impact the people we partner with so that they can access their own unique voice, range of healing choices, and their innate right to healthy power, we must first understand who a person is, “how they think, what they believe, and how they define wellness” (39, p. xxi). It is the practitioner’s duty to research which types of counseling and healing interventions are considered effective by the individuals with whom they work. All cultures have their belief systems, theories of wellness and illness, and ways of explaining problematic behaviors; these are considered Ways of Knowing. They also have their own healing methods and methodologies, known as Ways of Healing (2).

### Traditional indigenous knowledge, salutogenesis, and mind–body medicine

**Figure 1:**
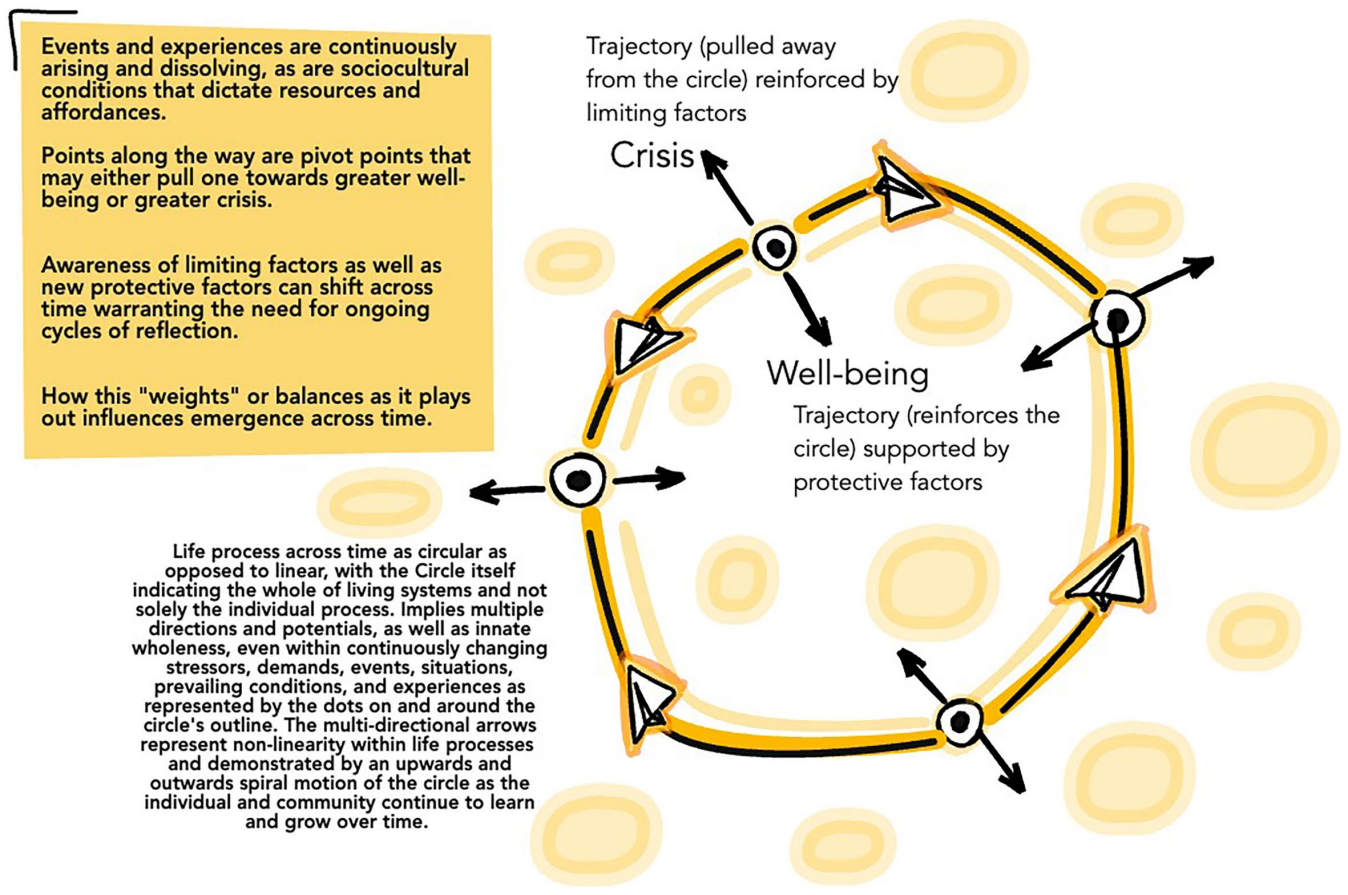
Description: indigenous view of salutogenesis.

Antonovsky’s salutogenic theory presented the concepts of sense of coherence (SOC) and generalized resistance resources (GRRs). SOC reflects a lens of interdependence that approaches individual lives as existing in intimate relationships with the world around them. An individual or community with well-developed SOC approaches life as meaningful, understandable, relatable, and navigable (40). GRRs (resilient factors) reflect the characteristics of individuals, families, communities, societies, and environments that mitigate tensions that otherwise adversely impact health and well-being. When GRRs are operating effectively, they reinforce SOC (41). Achieving the aim behind the concept of GRRs and SOC through the applications of mind–body medicine and integrative health can shift health trajectory away from greater crisis and toward the potential for greater well-being. This is achieved by focusing on protective factors, resources, and skills, as opposed to solely limiting factors and dynamics (42, 43). A working framework for Indigenized salutogenesis is presented in Figure 1 and reinforced by Tables 1, 2.

**Table 1:** Building from Grabowski et al.’s work on applying living systems theory to health promotion, parallels between four guiding principles, TIKCP, and the mind–body skills group model, are explored.

Principle	TIKCP parallels	MBSG parallels and tips
Promoting health is partially a process of sharing information. Such information will never be wholly understood in the form it is communicated; thus direct/complete transfer of health knowledge is impossible.	Inherent to the historical nature of talking circles, healing circles, community councils, or ceremonies is the stance of mutual respect that supported individual voices to inform the arrival at collectivist needs, answers, and decisions. Such a model is one of equality and reflects the Indigenous value that “within the circle, we all are of equal height” (unknown).	Offers scientific/health information (e.g., stress/trauma) with humility and non-attachment to how it is received, understood, and/or acted upon. Regular spaces for interpretive relay and feedback: “Is anyone willing to share if/how this information is/is not relevant or useful to you and your community?”.
Everyone observes and experiences their health, health-related information, and/or health behaviors in different ways than their peers; thus, any messaging will mean something different in the context of the individualized and layered environments (social, cultural, political, natural, built, organizational, etc.) that each person (and community) experiences.	Rites of passage, spiritual belief systems around individual community roles and destinies, and the importance of individual awareness, development, and responsibility to the collective. The latter reflects a different brand of self-care than in today’s world, wherein self-care aimed to serve community needs over individual posturing. I.e., self-care was less egocentric. Meditation practices in many forms arose inherent to Indigenous populations across disparate geographic regions.	Inherent to the collectivist-oriented design (contrasted to 1:1 pathogenic-focused models) is safe development of self-referential processes; in relationship to others. This design combines with skills that support capacity to understand self/other better. While theoretical, it is proposed that there is an amplifying effect to this process by being in a group (collectivist-minded) setting. Lastly, principles of trauma-informed spaces support reclamation and development of agency in a setting of relational support.
Meaning will be more/less established as individual self-understanding that defines how/when a health promoting environment is seen as more/less meaningful; the individual’s sense of health-related meaning determines what is deemed relevant. I.e., all meaning is self-defined. How education impacts health is fundamentally unpredictable.	Many Indigenous cultures carry beliefs that individuals are born into specific dispositions, callings, and roles in the community. For example, a person exhibiting a state that western mental health would pathologize, is seen as carrying medicine that serves the people. As such, containers were held for concurrent and interactional self- and community-oriented meaning.	Intentional design of spaces that support inclusion and safe exploration of individual and group awareness in non-advising, non-pathologizing, and non-fixing ways. Scientific education presented as flexible, changing, and valuable if relevant to lived experience.
For health promotion initiatives to be meaningfully understood, they need to fit into existing sociocultural structures that are woven into relevant persons, roles, or relational constructs. Therefore, it is essential for communication to meet expectations if utility is to be realized.	Upholds that all manifestations are interdependent, including within and between individuals and their multi-faceted environments. Trust in a larger web of learning processes that play out in individuals, groups, and the collective. What one does carries influence, though possibly unseen at the time, to the entire web.	Supports and encourages learners to interpret and adapt any element of the approach to their understanding of self, community, or culture for enhanced accessibility utility across disparate geographies, environments, and populations.

**Table 2:** An exploration of differences between TIKCP and colonized/westernized approaches to various sociocultural topics, views, values, approaches, and domains.

Paradigm: Topic:	TIKCP	Westernized
Cultural psychology	Prioritizes group harmony and sociocultural needs. Values vulnerability, mutual responsibility, authenticity, and courage. Embraces uncertainty, curiosity, imperfection, cultural and epistemic humility. Pluralism—acknowledges multiple truths and realities. Flux—embraces emergence of new ideas and identities.	Prioritizes personal goals, individual needs, self-sufficiency, and grit. Values certainty, confidence, masking authenticity; may be underpinned by inadequacy. Drives performative perfectionism. Humility seen as a weakness, may lack accountability. Singularity of view that privileges certain cultures or contexts. Presumes static/fixed states/identities and objectivity.
Resources	Principle of communal sufficiency where there is enough for all to live meaningful, healthy, and productive lives, including in relationship to others and the natural and spiritual world, which are seen as interwoven. Stewardship-oriented.	Focus on limitation of resources, proprietary tendencies in individuals and organizations. Competition, productivity, accumulation. Unchecked extractivism and minimization of ecosystem impacts. Ownership-focused.
Relational accountability	Authentic connection. Aims toward unconditional care. Mutual respect and community support for individual differences. Recognizes interdependence between individuals and actions. Trust-focused, collaboration, cooperation. Values responsibility-generativity. Prioritizes relationship over approach.	Treats people as separate. Transactional in nature (i.e., what do I get out of this). Diminished attention toward acknowledging how individual choice and actions impact others, the collective, and/or the ecological and environmental levels. Competitive; may breed distrust. Prioritizes approach over relationship.
Self-care	Tending to self is within the context of community needs; individual healing concurrently supports addressing social determinants of health.	Focused on feeling good, individualized concepts of success, and/or happiness; in some cases, may bypass depth-oriented inner work, and/or personal responsibility for the impact of one’s actions on others or the layered environment.
Knowledge/ways of knowing	Encompasses embodied and intuitive ways of knowing in addition to cognitive/ intellectual. Acknowledges centers of knowing in the heart, gut, and womb, in addition to the head/brain. Values oral tradition and storytelling as a means of knowledge preservation.	Focused on cognition and intellect, “hard”-data driven. Decreased valuation of oral tradition/ storytelling as a means of knowledge transmission. Content focused which may miss larger contexts including those that that may be hidden from the conscious mind.
Power/leadership	Power *with*, equality based. Leader as advocate for highest benefit of all. Leader is someone who others respect and follow because of their values-based reciprocal behaviors.	Power *over*, domination based. Leader as authority over others. Leader viewed as one who has “earned the right” through educational attainment and title/role progression.
Space	Opening space. Expanding choice. Values and invites curiosity, contemplation, and psychological flexibility. Proactively inclusive. Consistent unbiased spaces of support.	Closing space. Removing choice. Favors certainty and decisiveness. Imposes change and passively or actively excludes participation in proprietary ways.
Spirituality	Recognizes a living spirit in all things. Acknowledges interconnection amongst all beings/things, seen/unseen, material/spiritual. Embraces impermanence and cycles of life. Consistent consideration and engagement with kinship, ritual, and ceremony.	Individually chosen belief systems. Duality, right/wrong. Fosters othering. Cultivates avoidance of existential realities that carry potential to temper human tendencies that reinforce suffering. Inclined toward separating the spiritual domain from other elements of life and health.
Health/healing systems	Strength-focused. Holism. Circular, spiral, and non-linear processes. Healing-centered engagement. Community-involved care seeks to remedy collectivist imbalance. Engages culture as a protective and reparative health factor. Being with vs. doing to another. Integration with the natural world.	Deficits-focused. Reduction to parts. Linear views/approaches. Pathologizing. Symptom masking/remediation. May fail to adequately address social determinants of health, environmental, and other root factors. Doing to vs. being with and attunement to other.
Relational ecology	Embraces an intimate relationship with land, nature, and the whole of the environment as sacred, interdependent, and fundamental in healing processes.	Separated from nature/environmental influences on health and well-being.
Technology, economy, politics, and/or education	Values-driven action. TIKCP supports modern innovation and technology—e.g., solutions to energy, natural resources, and agriculture. Mutual learning and creativity are valued. Open systems, values space for inquiry. Social justice oriented.	Science-focused intertwined with capitalism-driven action. Separates wisdom traditions and spirituality from other domains. Top-down learning. Stifling of creativity. Encouragement of obedience. Decreased focus on social justice. Restraint/exclusion of other forms of knowledge or belief systems.
Gender and sexuality	Embraces a spectrum, approaches difference as sacred, honored with spiritual interpretations and roles.	Binary, dualistic. Minoritizes and/or marginalizes difference. Shame-based.

In Antonovsky’s work, three components inherent to SOC were described as (a) comprehensibility—a sense of structure, order, consistency, clarity, or predictability; (b) manageability—a belief that one has the resources to relate and respond to demands; and (c) purpose and meaning—a framework that stressors tie into larger forces, patterns, and that how one relates and responds to what manifests, carries impact or influence upon the larger order of things (40). First and foremost, we suggest that TIKCP has innately carried these components across time and generations of immeasurable adversity. Second, we believe the underlying design and intention inherent to the MBSG model—when delivered in a way that is true to these aims, supports strengthening synergistic relationships between GRRs and SOC at individual and collectivist levels.

Going further and building from the work of Grabowski (44) and Grabowski et al. (45) on the application of systems theory to health promotion, four principles (Table 1) must be understood to underscore uncertainty when attempting to transfer health-promoting models. These principles are presented with statements that reflect proposed alignment between salutogenesis, living systems theory, TIKCP, and the MBSG approach.

## Detail

### Community-centered re-storying of mind–body medicine

Re-storying “revisits and recuperates in order to restore” (46, p. 4) a new sense of possibility through the understanding of what Indigenous cultures have been practicing for millennia. The essence of re-storying, which some frame as neurodecolonizing (47) and others as indigenizing (48, 49), is to elevate voices that both can provide new and innovative suggestions. This is attained by honoring and learning from the wisdom of our elders and ancestors to bring forth practical and impactful change within Indigenous communities. The preceding section has begun to paint this picture. Re-storying is accomplished by using strategies that intertwine past, present, and future narratives into meaningful movement toward new possibilities. Re-storying is a dynamic, participatory, and reciprocal process among those disseminating salutogenic-oriented models. This process directly engages the individuals, cultures, and communities who are invited to examine their utility, including those receiving training in the approach alongside community recipients of delivered programs.

An example of optimizing delivery of salutogenic approaches in diverse sociocultural landscapes can be seen in the effort to examine, understand, and optimize cultural integration of models such as the MBSG approach in Indigenous communities. Within this context, several questions are essential to pose: (1) Is integration optimal? and (2) How does aiming to support the integration of contemporary westernized models of science and delivery with TIKCP benefit those living in our current world (50)?

To address these questions, it is critical that the rationale, design, and content of health promotion programs be brought forth to any given community as an inquiry and invitation. The stance for approaching this must be rooted in healing-centered and strength-based orientations of reciprocity, providing true opportunities for co-development of programming that counters deficit-focused or unintentional rescuer roles that inadvertently reinforce dynamics of racialization or minoritization. For Indigenous populations, to reclaim and effectively re-story the MBSG model and bring it into optimal alignment with TIKCP, Indigenous practitioners, grounded in who they are and while continuing to deepen their own healing, weave their values, stories, songs, traditions, and rituals into the approach to enrich and tailor it for the relatives they serve (51, 52). Examples of adaptations to design and delivery include the integration of smudging, drumming, prayer, adaptations to movement practices, and customized skill delivery such as meditation or guided imagery scripts. These practices are integrated with the guidance of community-identified leaders, elders, healers, and culture bearers to determine what elements of the base design or other modifications best centered the values and needs of their community.

Areas of distinction between westernized and TIKCP characteristics that are important to consider in the re-storying of salutogenic models, especially within the structures and dynamics of organizations and systems that are disseminating them, are summarized in Table 2 (17, 53–58). As previously encouraged, reviewing the Table with non-dualistic flexibility supports the need for balance within the realities of contemporary life. Those working in fields linked to salutogenesis, integrative health, and health promotion, are encouraged to explore the specific relevance of these elements in their work including from the understanding that health cannot be separated from prevailing social, economic, environmental, and cultural conditions. This invitation offers truly integral approaches that avoid the trap of colonial and neoliberal interpretations and abuses (e.g., deflection of responsibility away from systems and organizations). Since system change is slow, repeated acknowledgement and advocacy serve as the impetus for enactivism, emergence, and embodiment of strategies that effectively leverage the theoretical constructs of salutogenesis.

## Discussion

The reclamation and application of TIKCP in its many variations across the globe is an important yet uncertain task. At a minimum, and despite a gap between the theoretical acknowledgment of cultural factors in health and the practical outcomes observed in communities (59), cultural reclamation and community sovereignty must be seen as one of the most critical of healing elements; this need is greater in some communities than others. Cultural restoration and integration applied to the design and deployment of salutogenic models benefits from understanding and being responsive to three levels (60) of relevance: the health (stability, consistency) *of* the culture (61), culture *as a social determinant* of health (62), and *culture-as-health* which involves Indigenous ways of knowing, cultural practices, place-based/land/sacred sites, and spirituality (63), all of which we consider innate within TIKCP.

Approaching salutogenic models from the lens of TIKCP carries potential to improve contemporary problem-solving skills for various communities and the implicit cultures that inform them as living systems. This approach serves to support the maintenance of spiritual practices, languages, and cultural preservation. We argue that the potential utility of the salutogenic model is amplified for Indigenous populations by recognizing the sacred and historical origins of the concepts as not new or unique to their contemporary form. Further, re-storying and culturally aligning the model, place by place and directly by the Indigenous persons it aims to serve, is a critical and necessary strategy. This effort must apply when deploying salutogenic and integrative health models into any population, as implicit assumptions may carry forward, adding harm.

Given that the lived experience that informs this community study arises predominantly from North American Indigenous populations, gaps that improve the extendability of these principles and suggestions may exist. At the same time, a generalized and adaptable framework in support of approaching any globally dispersed Indigenous population with a healing or health promotion model is presented. Future scholarship that presents unique experiences in other populations is encouraged. Aligning the presented concepts with the reality of modern health care and economic systems, and prevailing sociocultural dynamics, requires an ongoing creative co-creation amongst stakeholders. Health care systems, policy makers, and service organizations must be at the forefront of these efforts by critically evaluating their roles and proactively serving the transformational process.

Past work has presented concerns around the potential for “colonial reproduction” that may emerge when TIKCP is integrated into western pedagogical contexts (64). In support of inclusive, equitable, and culturally safe salutogenic models/environments, the lessons learned from this process of re-storying a contemporary approach invite caution for anyone engaging Indigenous or other communities historically devalued or targeted by discrimination. Key areas of attention include (a) honoring and recognition of the population’s unique history including cultural and spiritual traditions and strengths; (b) responsible co-creation; (c) humility, respect, and a non-defensive willingness to listen and learn; (d) non-hierarchical learning and organizational structures, especially among leadership teams; (e) embodying what supports safety in individual and/or group settings; (f) trauma sensitivity and responsiveness; (g) full permission to adapt the approach to cultural and spiritual frameworks in support of the reclamation of Indigenous healing practices; (h) encouraging Native language integration; and (i) a lens that no one “owns” these practices and teachings but that everyone is responsible for protecting and honoring them.

The described elements of the approach are not to dissuade attempts to share them, but rather for them to be deployed using a respectful and responsible community-based participatory action approach, without interference in the community’s choices and strategies for alteration and use. Such an approach increases integrity in the integration of holistic methodologies for greater salutogenic benefit for all (65–67).

Ultimately, re-storying Indigenous health is achieved through “the search, not the answer; by remaining open to new inspirations, opportunities, and possibilities; and by knowing that sustaining the economic development of Indigenous communities means never assuming the story is over” (46, p. 2). By employing an Indigenous-led, healing centered engagement within a peer-to-peer support model that integrates modern neuroscience with the rich traditional knowledge of Indigenous healing methods, we can enhance accessibility and promote interdependent, reciprocal sustainability.

## Data Availability

The original contributions presented in the study are included in the article/supplementary material, further inquiries can be directed to the corresponding author.

## References

[1] Evering B. Relationships between knowledge(s): implications for ‘knowledge integration’. J Environ Stud Sci. (2012) 2:357–68. doi: 10.1007/s13412-012-0093-9

[2] Olson KR. Inviting the sacred wound into circle: Re-storying an indigenous mind-body medicine framework for healing. Bradenton, FL: Breyer State Theology University; (2025). Available online at: https://static1.squarespace.com/static/67d4781fa5ca580f490bd5c5/t/68e7dd0d0c2ff661d9525e37/1760025869195/FINAL+OLSON.KIMBER+PHD+DISSERTATION.pdf (Accessed September 1, 2025).

[3] Roher SIG Yu Z Martin DH Benoit AC. How is Etuaptmumk/two-eyed seeing characterized in indigenous health research? A scoping review. PLoS One. (2021) 16:e0254612. doi: 10.1371/journal.pone.0254612, 34283831 PMC8291645

[4] Lindström B Eriksson M. Contextualizing salutogenesis and Antonovsky in public health development. Health Promot Int. (2006) 21:238–44. doi: 10.1093/heapro/dal016 16717056

[5] Srivastava P. "Why is epistemic humility provocative? A reflexive story" In: Faul MV, editor. Transforming development in education. Cheltenham, UK Northampton, MA: Edward Elgar Publishing (2025) 157–74. doi: 10.4337/9781035337798.00017

[6] Tervalon M Murray-García J. Cultural humility versus cultural competence: a critical distinction in defining physician training outcomes in multicultural education. J Health Care Poor Underserved. (1998) 9:117–25. doi: 10.1353/hpu.2010.0233, 10073197

[7] Willis D Backes E. Early relational health: Building foundations for child, family, and community well-being. (Committee on the Early Relational Health Determinants of Future Health and Well-Being Reports and Resources). National Academies. (2025). Available online at: https://nap.nationalacademies.org/read/29234/chapter/1 (Accessed October 15, 2025). 41529129

[8] Zanchetta MS Stevenson M Nenadovic V Perreault M Henry CJ Leong N. An Emancipating-salutogenesis conceptual framework & model of Anishinaabe balance promotion for health. Aporia. (2016) 8:16–30. doi: 10.18192/aporia.v8i2.2792

[9] Wold B Mittelmark MB. Health-promotion research over three decades: the social-ecological model and challenges in implementation of interventions. Scand J Public Health. (2018) 46:20–6. doi: 10.1177/1403494817743893, 29552963

[10] Braveman P Gottlieb L. The social determinants of health: it's time to consider the causes of the causes. Public Health Rep. (2014) 129:19–31. doi: 10.1177/00333549141291S206, 24385661 PMC3863696

[11] Nicholls D Gibson BE. Physiotherapy otherwise. Aotearoa: New Zealand: Tuwhera Open Books, Auckland University of Technology, Tämaki Makaurau. (2022) doi: 10.24135/TOAB.8

[12] Flynn MB. Global capitalism as a societal determinant of health: a conceptual framework. Soc Sci Med. (2021) 268:113530. doi: 10.1016/j.socscimed.2020.113530, 33288355

[13] Alang S Hardeman R Karbeah J Akosionu O McGuire C Abdi H . White supremacy and the Core functions of public health. Am J Public Health. (2021) 111:815–9. doi: 10.2105/AJPH.2020.306137, 33826395 PMC8033999

[14] Poirier B Sethi S Haag D Hedges J Jamieson L. The impact of neoliberal generative mechanisms on indigenous health: a critical realist scoping review. Glob Health. (2022) 18:61. doi: 10.1186/s12992-022-00852-2, 35705995 PMC9199313

[15] Fries CJ. Governing the health of the hybrid self: integrative medicine, neoliberalism, and the shifting biopolitics of subjectivity. Health Sociol Rev. (2008) 17:353–67. doi: 10.5172/hesr.451.17.4.353

[16] Fries CJ. Healing health care: from sick care towards Salutogenic healing systems. Soc Theory Health. (2020) 18:16–32. doi: 10.1057/s41285-019-00103-2, 32226316 PMC7099730

[17] Keikelame MJ Swartz L. Decolonising research methodologies: lessons from a qualitative research project, Cape Town, South Africa. Glob Health Action. (2019) 12:1561175. doi: 10.1080/16549716.2018.1561175, 30661464 PMC6346712

[18] Gordon JS. Mind-body skills groups for medical students: reducing stress, enhancing commitment, and promoting patient-centered care. BMC Med Educ. (2014) 14:198. doi: 10.1186/1472-6920-14-198, 25245341 PMC4181427

[19] Gordon JS Staples JK He DY Atti JAA. Mind–body skills groups for posttraumatic stress disorder in Palestinian adults in Gaza. Traumatology. (2016) 22:155–64. doi: 10.1037/trm0000081

[20] Aalsma MC Jones LD Staples JK Garabrant JM Gordon JS Cyr LR . Mind-body skills groups for adolescents with depression in primary care: a pilot study. J Pediatr Health Care. (2020) 34:462–9. doi: 10.1016/j.pedhc.2020.05.003, 32861425

[21] Kelly L Rowe C Choudhury A Woo-Cater S Greenwood L. Evaluating a peer-support mind-body medicine intervention for healthcare leaders. Worldviews Evid-Based Nurs. (2024) 21:626–33. doi: 10.1111/wvn.12750, 39431561

[22] Payne D Olson K Parrish JW. Pathway to Hope: an indigenous approach to healing child sexual abuse. Int J Circumpolar Health. (2013) 72:72. doi: 10.3402/ijch.v72i0.21067, 23984282 PMC3753130

[23] Drywater-Whitekiller V. Integrated curriculum guide for social work practice with American Indians and Alaska natives in child welfare. National Child Welfare Workforce Institute (NCWWI). (2024). Available online at: https://ncwwi.org/wp-content/uploads/2024/05/Integrated-Curriculum-Guide-for-Social-Work-Practice-With-American-Indians-and-Alaska-Natives-in-Child-Welfare.pdf (Accessed August 24, 2025).

[24] Brave Heart MYH Chase J Elkins J Altschul DB. Historical trauma among indigenous peoples of the Americas: concepts, research, and clinical considerations. J Psychoactive Drugs. (2011) 43:282–90. doi: 10.1080/02791072.2011.628913 22400458

[25] Brockie TN Dana-Sacco G Wallen GR Wilcox HC Campbell JC. The relationship of adverse childhood experiences to PTSD, depression, poly-drug use and suicide attempt in reservation-based native American adolescents and young adults. Am J Community Psychol. (2015) 55:411–21. doi: 10.1007/s10464-015-9721-3, 25893815

[26] Spillane NS Schick MR Kirk-Provencher KT Nalven T Goldstein SC Crawford MC . Trauma and substance use among indigenous peoples of the United States and Canada: a scoping review. Trauma Violence Abuse. (2023) 24:3297–312. doi: 10.1177/15248380221126184, 36197078 PMC12109140

[27] Walters KL Mohammed SA Evans-Campbell T Beltrán RE Chae DH Duran B. BODIES DON’T JUST TELL STORIES, THEY TELL HISTORIES: embodiment of historical trauma among American Indians and Alaska natives. Du Bois Rev. (2011) 8:179–89. doi: 10.1017/s1742058x1100018x, 29805469 PMC5967849

[28] Nartey J. Deforestation and the Erosion of indigenous healing: the impact of ecological degradation on medicinal plant biodiversity and traditional health systems. SSRN.(2025). Available online at: https://ssrn.com/abstract=5242586

[29] Barnes P Adama P Powell-Griner E. Health characteristics of the American Indian or Alaska native adult population: United States, 2004–2008. Center for Disease Control. Report No.: 20; (2010) p. 1–24. Available online at: https://www.cdc.gov/nchs/data/nhsr/nhsr020.pdf (Accessed November 13, 2025) 20583451

[30] Plani F Carson P. The challenges of developing a trauma system for indigenous people. Injury. (2008) 39 Suppl 5:S43–53. doi: 10.1016/S0020-1383(08)70028-7, 19130917

[31] Zuckerman S Haley J Roubideaux Y Lillie-Blanton M. Health service access, use, and insurance coverage among American Indians/Alaska natives and whites: what role does the Indian Health Service play? Am J Public Health. (2004) 94:53–9. doi: 10.2105/AJPH.94.1.53, 14713698 PMC1449826

[32] Henderson A MacLehose RF Manson SM Buchwald D. Social determinants of health, tribal payments, and probability of contracting COVID-19 in American Indian and Alaska native peoples. Int J Soc Determinants Health Health Serv. (2025) 55:46–54. doi: 10.1177/27551938241277130, 39155571

[33] Gurven MD. Broadening horizons: sample diversity and socioecological theory are essential to the future of psychological science. Proc Natl Acad Sci USA. (2018) 115:11420–7. doi: 10.1073/pnas.1720433115, 30397108 PMC6233064

[34] Henrich J Heine SJ Norenzayan A. The weirdest people in the world? Behav Brain Sci. (2010) 33:61–83; discussion 83-135. doi: 10.1017/s0140525x0999152x, 20550733

[35] Deshpande V Tinlin-Dixon R Java S Deshpande S. ‘An exploration of self (to other and to self) through therapist–client interactions’: trainee CAT practitioners’ reflections on personal therapy during training. Couns Psychother Res. (2025) 25:e70051. doi: 10.1002/capr.70051

[36] Walsh R. What is wisdom? Cross-cultural and cross-disciplinary syntheses. Rev Gen Psychol. (2015) 19:278–93. doi: 10.1037/gpr0000045

[37] Halifax J. A heuristic model of enactive compassion. Curr Opin Support Palliat Care. (2012) 6:228–35. doi: 10.1097/spc.0b013e3283530fbe, 22469669

[38] Hussain Z Khan S Ahmed N Arif S Naeem A. Concept analysis on cultural humility. JPEHSS. (2025) 3:184–9. doi: 10.63163/jpehss.v3i2.330

[39] Reimers DM. Unwelcome strangers: American identity and the turn against immigration. New York, NY: Columbia University Press (1999). 199 p.

[40] ANTONOVSKY A. The salutogenic model as a theory to guide health promotion. Health Promot. J Int. JSTOR (1996) 11:11–8. doi: 10.1093/heapro/11.1.11

[41] Antonovsky A. Health, stress, and coping. San Francisco: Jossey-Bass (1979).

[42] Erb M Winkle D. "Defining the need" In: Erb M Schmid AA, editors. Integrative rehabilitation practice: The foundations of whole-person Care for Health Professionals. London, United Kingdom: Singing Dragon, Jessica Kingsley (2021). 57–73.

[43] Low M. A novel clinical framework: the use of dispositions in clinical practice. A person centred approach. J Eval Clin Pract. (2017) 23:1062–70. doi: 10.1111/jep.12713, 28220638

[44] Grabowski D. Health identity: theoretical and empirical development of a health education concept. JSR. (2015) 6:141–157. doi: 10.5296/jsr.v6i1.7754

[45] Grabowski D Aagaard-Hansen J Rod MH Jensen BB. Complexity theory in health promotion research: four essential principles based on Niklas Luhmann’s systems theory. Societies. (2023) 13:253. doi: 10.3390/soc13120253

[46] Voyageur CJ Calliou B Brearley L. Restorying indigenous leadership: Wise practices in community development. 2nd ed. Banff: Banff Centre Press (2015). 345 p.

[47] Gray M. Decolonizing social work. Burlington: Ashgate (2013).

[48] Pidgeon M. More than a checklist: meaningful indigenous inclusion in higher education. SI. (2016) 4:77–91. doi: 10.17645/si.v4i1.436

[49] Gaudry A Lorenz D. Indigenization as inclusion, reconciliation, and decolonization: navigating the different visions for indigenizing the Canadian academy. AlterNative. (2018) 14:218–27. doi: 10.1177/1177180118785382

[50] Duran E. Healing the soul wound: Trauma-informed counseling for indigenous communities. 2nd ed. New York: Teachers College Press (2019).

[51] Meyerhoefer T Riegert J Allerton E Ranjbar N. Mindfulness has parallels to indigenous cultural practices. PN. (2022) 57:appi.pn.2022.11.11.28. doi: 10.1176/appi.pn.2022.11.11.28

[52] Smith-Yliniemi J Malott KM Riegert J Branco S. Utilizing collective wisdom: ceremony-assisted treatment for native and non-native clients. Prof Counsel. (2024) 13:448–61. doi: 10.15241/jsy.13.4.448

[53] Vaandrager L Kennedy L. "The application of salutogenesis in communities and neighborhoods" In: The handbook of salutogenesis. New York: Springer (2017). 159–70. 28590643

[54] Sweet MA Dudgeon P McCallum K Ricketson MD. Decolonising practices: can journalism learn from health care to improve indigenous health outcomes? Med J Aust. (2014) 200:626–7. doi: 10.5694/mja14.00528, 24938333

[55] Whiting L Kendall S Wills W. An asset-based approach: an alternative health promotion strategy? Commun Pract J Commun Pract Health Visitors’ Assoc. (2012) 85:25–8.

[56] Wilson D Neville S. Culturally safe research with vulnerable populations. Contemp Nurse. (2009) 33:69–79. doi: 10.5172/conu.33.1.69, 19715497

[57] Moodley K Singh S. “It’s all about trust”: reflections of researchers on the complexity and controversy surrounding biobanking in South Africa. BMC Med Ethics. (2016) 17:57. doi: 10.1186/s12910-016-0140-2, 27724893 PMC5057490

[58] Gosnell F McKergow M Moore B Mudry T Tomm K. A galveston declaration. J Syst Ther. (2017) 36:20–6. doi: 10.1521/jsyt.2017.36.3.20

[59] Biles BJ Serova N Stanbrook G Brady B Kingsley J Topp SM . What is indigenous cultural health and wellbeing? A narrative review. Lancet Reg Health West Pac. (2024) 52:101220. doi: 10.1016/j.lanwpc.2024.101220, 39664592 PMC11632815

[60] Benz C Bull T Mittelmark M Vaandrager L. Culture in salutogenesis: the scholarship of Aaron Antonovsky. Glob Health Promot. (2014) 21:16–23. doi: 10.1177/1757975914528550, 24814861 PMC4242901

[61] Thorpe A Yashadhana A Biles B Munro-Harrison E Kingsley J. Indigenous health and connection to country. (2023). Available online at: 10.1093/acrefore/9780190632366.013.436 (Accessed January 31, 2023)

[62] Verbunt E Luke J Paradies Y Bamblett M Salamone C Jones A . Cultural determinants of health for aboriginal and Torres Strait islander people - a narrative overview of reviews. Int J Equity Health. (2021) 20:181. doi: 10.1186/s12939-021-01514-2, 34384447 PMC8359545

[63] Yamane CYEW Helm S. Indigenous culture-as-health: a systematized literature review. J Prev Dent. (2022) 43:167–90. doi: 10.1007/s10935-022-00666-3, 35286545

[64] St. Denis V. Silencing aboriginal curricular content and perspectives through multiculturalism: “there are other children here.”. Rev Educ Pedagog Cult Stud. (2011) 33:306–17. doi: 10.1080/10714413.2011.597638

[65] Persaud JN Wannamaker K Stark K Lambert C Harrison C Keller N. Decolonizing education: advancing indigenous student success through culturally responsive practices in Ontario. AlterNative. (2025) 21:264–73. doi: 10.1177/11771801251340657

[66] Denichaud D. In support of pedagogical Salutogenesis: Exploring holistic, traditional, and indigenous health methodologies toward an ethic of (self/school) care. University of Toronta. (2020). Available online at: from: http://hdl.handle.net/1807/100894

[67] Michaelson V Pickett W Davison C. The history and promise of holism in health promotion. Health Promot Int. (2019) 34:824–32. doi: 10.1093/heapro/day039, 29897526

